# Transvaginal ultrasound-detected endometrial echogenicity heterogeneity in diagnosing endometrial carcinoma: risk factors and nomogram-based prediction model

**DOI:** 10.3389/fonc.2026.1682386

**Published:** 2026-03-06

**Authors:** Ling Yan, Jianxia Sun, Shengping Yang, Dingyi Wang, Xiaowen Zuo, Mingming Zhang, Can Zhang, Ting Zhang, Huaping Jia

**Affiliations:** 1 Department of Ultrasound Diagnosis, The Ninth Medical Center of Chinese PLA General Hospital, Beijing, China; 2 Department of Obstetrics, Heze Maternal and Child Health Hospital, Heze, China

**Keywords:** diagnosis, endometrial cancer, endometrial echogenicity, risk factors, transvaginal ultrasound

## Abstract

**Objective:**

This study aimed to develop a nomogram prediction model based on risk factors associated with endometrial cancer (EC) diagnosed via transvaginal ultrasound (TVS)-detected non-uniform echogenicity.

**Methods:**

A retrospective analysis of 564 female patients (control group: normal/benign lesions, n = 475; observation group: EC, n = 89) was conducted. TVS findings were compared with pathological diagnoses, and receiver operating characteristic (ROC) analysis was performed to assess diagnostic performance. Patients were split 7:3 into training and internal validation sets. Multivariate logistic regression identified predictors for nomogram construction, which was validated for performance and utility. SHAP (SHapley Additive exPlanations) analysis was applied for model interpretability, and clinical cases were used for demonstration.

**Results:**

The area under the curve (AUC) of TVS detection of endometrial echogenicity heterogeneity for EC diagnosis was 0.726. Multivariate logistic regression analysis showed that body mass index (BMI), hypertension, diabetes, age at menopause > 50 years, and non-uniform echogenicity were risk factors for EC. The prediction model constructed demonstrated good calibration performance in the training set and excellent discrimination ability and stable predictive consistency in the internal validation set. Decision curve analysis further confirmed its clinical utility. SHAP analysis of the established nomogram revealed that age at menopause and heterogeneous endometrial echogenicity were the most influential predictors in the model, with echogenicity heterogeneity consistently associated with an increased risk of EC. When the nomogram predicted an EC probability of ≥ 0.5, the number of predicted positive cases was 93 (16.49%), showing no statistically significant difference (*P* > 0.05) from the 89 actually confirmed EC cases (15.78%). This indicates a high agreement between model predictions and actual outcomes.

**Conclusion:**

TVS detection of heterogeneous endometrial echogenicity holds supplementary diagnostic value for EC. The nomogram model constructed in this study integrates key clinical and sonographic features, demonstrating favorable predictive performance and clinical applicability. SHAP analysis confirmed that echogenicity heterogeneity and age at menopause are important predictors, enhancing the model’s interpretability. This tool aids in early identification of high-risk patients and provides a reference for clinical decision-making.

## Introduction

Endometrial cancer (EC) is one of the most common malignant tumors of the female reproductive system, with an incidence that is rising year by year, especially among postmenopausal women (1). Related data show that EC has the highest number of new cases among gynecological malignancies each year (2). The early symptoms of EC are usually subtle and similar to those of other gynecological diseases (such as endometrial hyperplasia and polyps) (3). Therefore, early diagnosis is challenging but crucial for improving the prognosis of patients with EC.

At present, the diagnosis of EC mainly relies on tissue biopsy or hysteroscopy. However, these methods have limitations such as invasiveness, high cost, and complex operation, making them unsuitable for large-scale screening (4). In contrast, TVS has been widely used in the screening of gynecological diseases due to its advantages of being non-invasive, rapid, cost-effective, and easy to operate. More importantly, studies have confirmed that assessing endometrial echogenicity heterogeneity through TVS has clear clinical value for the early diagnosis of EC (5). This imaging feature is closely related to endometrial hyperplasia, polyps, and malignant lesions (6), and is a current research focus in the early diagnosis of EC, providing an important direction for breaking through the dilemma of early EC diagnosis. In addition to imaging features, the occurrence of EC is also related to a variety of risk factors, including hormone imbalance, gene mutation, obesity, diabetes, hypertension, and physiological factors such as early menarche and delayed menopause (7, 8). These factors may be involved in disease occurrence through different mechanisms, yet existing studies have insufficiently explored the combined predictive efficacy of these factors and endometrial echogenicity heterogeneity detected by TVS, and no integrated early diagnostic tools suitable for widespread clinical application have been developed yet.

Therefore, this study aims to systematically evaluate the value of endometrial echogenicity heterogeneity detected by transvaginal ultrasound in the diagnosis of EC, and to clarify the synergistic effect of this imaging indicator with clinical risk factors, and to construct a predictive model based on a nomogram in combination with relevant risk factors, in order to provide a reference for the early diagnosis of EC and clinical decision-making, and ultimately improve the treatment outcomes and quality of life of patients.

## Methods

### Study population

This study retrospectively analyzed the clinical data of 564 female patients who attended the Ninth Medical Center of the General Hospital of the People’s Liberation Army of China from January 2021 to January 2024. These patients presented with symptoms such as abnormal vaginal bleeding, irregular menstruation, or postmenopausal bleeding, and all underwent vaginal ultrasonography. The patients were divided into a control group (normal endometrium or benign lesions, n = 475) and an observation group (EC patients, n = 89) based on pathological findings.

Inclusion criteria: The observation group was diagnosed as having EC through preoperative endometrial biopsy and histological examination. The control group was also diagnosed as having normal or benign endometrial lesions through pathological examination. Moreover, the pathological results and clinical data of both groups were complete (9); Before admission, neither group of patients had received radiotherapy, chemotherapy, endocrine, or immunotherapy; All patients in the two groups were free from hematological disorders and did not have any severe organ dysfunction diseases affecting the heart, liver, or kidneys.

Exclusion criteria: those with combination of secondary tumors and other malignant tumors; those with severe medical diseases (heart failure, expiratory failure, etc.); those with severe hepatic and renal insufficiency, data insufficiency, and cognitive dysfunction; those with congenital anomalies of the reproductive system; and those with the presence of severe infections.

### Ethical considerations

This study was approved by the Ethics Committee of The Ninth Medical Center, Chinese PLA General Hospital (No. LL-LCSY-2025-02). All methods were carried out in accordance with Declaration of Helsinki. Given the retrospective nature of the study, the requirement for informed consent was waived by the institutional ethics committee. In accordance with the journal’s guidelines, we will provide our data for independent analysis by a team selected by the Editorial Team for the purposes of additional data analysis or for the reproducibility of this study in other centers if such is requested.

### Detection methods

**Figure 1:**
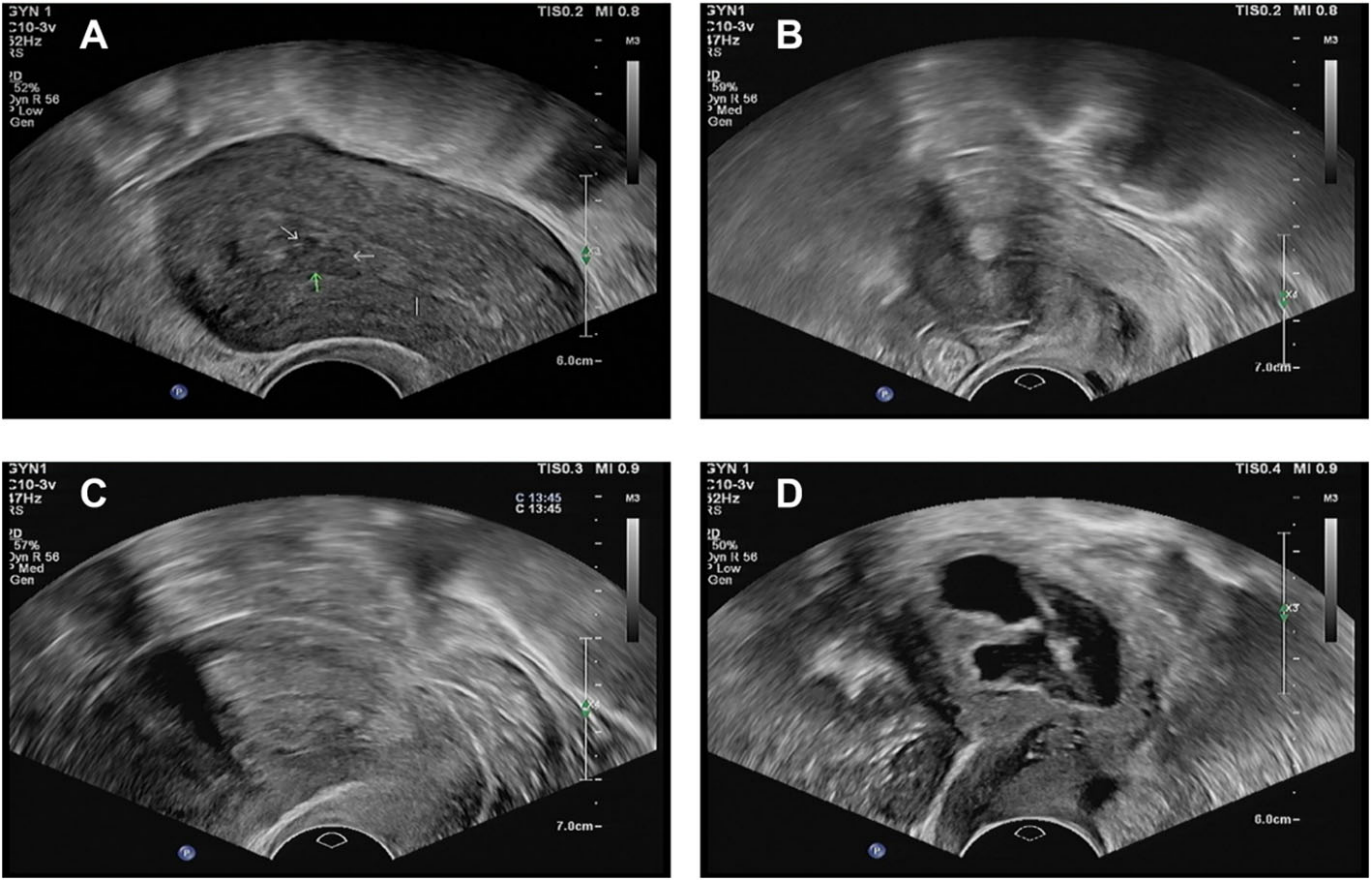
Ultrasonographic images of non-uniform endometrial echogenicity. **(A)** Focal non-uniform echogenicity. The image reveals a well-defined focal area within the endometrium exhibiting non-uniform echogenicity, contrasting with the relatively homogeneous surrounding endometrial background. This sonographic appearance is consistent with focal lesions, such as an endometrial polyp. **(B)** Focal non-uniform echogenicity (with cystic areas). The image demonstrates a focal, roundish lesion displaying characteristic non-uniform echogenicity, accompanied by anechoic cystic areas. The lesion is clearly demarcated from the myometrium. These findings suggest a focal endometrial lesion, such as an endometrial polyp. **(C)** Diffuse non-uniform echogenicity. The image shows diffuse thickening of the endometrium with entirely non-uniform echogenicity throughout the endometrial layer. The endometrial architecture appears disorganized with loss of homogeneity. No distinct focal mass is identified. This pattern of diffuse non-uniform echogenicity raises consideration for conditions such as diffuse endometrial hyperplasia. **(D)** Diffuse non-uniform echogenicity (with irregular background). The image depicts diffuse endometrial involvement characterized by non-uniform echogenicity, predominantly featuring a chaotic, heterogeneous background. The endometrial-myometrial junction is ill-defined. This sonographic presentation aligns with the features of diffuse endometrial pathology.

In this study, TVS was used as the main imaging evaluation method. All examinations were performed with Philips EPIQ 7 color Doppler ultrasound diagnostic instrument equipped with high frequency transvaginal cavity probe (frequency range: 3.0–10 MHz). Before the examination, the subject emptied the bladder and was placed in the lithotomy position of the bladder with full exposure of the vulva. The probe was covered with a sterile condom and applied with coupler. The probe was gently and slowly inserted into the vaginal vault by the operator. The following parameters were observed and recorded: (1) Endometrial thickness: the maximum thickness of bilayer endometrium (from the outer edge of the basal layer to the outer edge of the opposite side) was measured on the sagittal plane; (2) Endometrial Echogenicity Pattern: The uniformity of endometrial echogenicity was assessed and classified according to the International Endometrial Tumor Analysis (IETA) consensus terminology as follows (10, 11): Uniform Echogenicity: Defined as a continuous and homogeneous endometrial echo structure with symmetrical anterior and posterior layers. It includes the following subtypes: a) Triple-Layer Pattern: Typically observed in the late proliferative phase, characterized by a distinct hyperechoic midline and basal layer surrounding a hypoechoic functional layer; b) Uniform Hyperechogenicity: Commonly seen in the secretory phase or in postmenopausal endometrium, presenting as a uniformly hyperechoic endometrium; c) Uniform Isoechogenicity: Endometrial echogenicity similar to that of the myometrium; d) Uniform Hypoechogenicity: Endometrial echogenicity lower than that of the myometrium. Non-Uniform Echogenicity: Defined as the loss of homogeneous endometrial structure, presenting as heterogeneous, asymmetrical, or cystic changes, including the following features: a) Cystic Areas: Presence of one or more anechoic cystic structures within the endometrium, which may be regular or irregular in shape; b) Heterogeneous background: The entire endometrium exhibits disorganized echogenicity with loss of uniformity; c) Mixed Pattern: A heterogeneous background combined with regular or irregular cystic areas. A schematic diagram is provided in **Figure 1**. Static images and dynamic clips of all standard sections and abnormal areas were stored on the workstation and a preliminary report was generated. To ensure diagnostic accuracy, all images and reports were independently double-blind reviewed by two associate chief physicians with more than five years of experience in gynecological ultrasound, focusing on the above indicators. Disagreement on key features was referred to a senior expert with more than 10 years of experience in ultrasound diagnosis of gynecological oncology for arbitration. The arbitral expert, who was unaware of the initial opinion, reevaluated all images and made a final diagnosis based on the clinical data, which was used as the basis for research and analysis.

### Data collection

The clinical data and pathological results of the two groups were collected, including age, BMI, education level, residence, age of menarche, menopausal status, complications (hypertension, hyperlipidemia, diabetes, coronary artery disease), family history, hormone replacement therapy, endometrial thickness. All pathological specimens were processed according to standardized procedures. An experienced pathologist made the initial diagnosis, which was then independently reviewed and verified by a senior pathologist.

### Establishment and validation of the nomograms

This study aimed to develop and internally validate a nomogram for predicting EC risk. After randomly splitting the overall cohort at a 7:3 ratio (random-seed fixed) into a training set and an internal validation set, multivariable logistic regression was performed in the training set to identify independent predictors and to construct a quantitative nomogram. Systematic internal validation comprised three steps: (1) bootstrap resampling (1,000 repetitions) within the training set was used to plot a calibration curve and quantify overfitting; (2) the final model was applied to the separate validation set, where the area under the ROC was calculated for discrimination and a calibration curve was generated to assess agreement between predicted probabilities and observed outcomes; and (3) decision curve analysis (DCA) was conducted to estimate the clinical net benefit across a range of threshold probabilities. All statistical analyses were performed using the rms package (version 4.1.0) in R software.

### Statistical analysis

SPSS 27.0 and GraphPad Prism 8 were used for data analysis and graph drawing. Categorical variables are presented as frequencies and percentages, while continuous variables are expressed as medians or means ± standard deviations, depending on the data distribution. The Shapiro-Wilk test is used to assess the normality of the data. For continuous variables, one-way analysis of variance (ANOVA) is employed to compare demographic and baseline clinical characteristics across groups, whereas categorical variables are compared using the Pearson χ^2^ test. Additionally, the frequency of endometrial echogenicity heterogeneity in the observation and control groups is compared using the Pearson χ^2^ test to evaluate the diagnostic efficacy of TVS for detecting EC. The sensitivity, specificity, positive predictive value, negative predictive value, and diagnostic accuracy of heterogeneous echoes for diagnosing EC are also calculated to further assess their clinical value. Furthermore, ROC curves are plotted, and the AUC is calculated to quantitatively assess the diagnostic efficacy of TVS for detecting endometrial heterogeneity in EC. Variables with statistical significance (*P* < 0.05) in univariate analysis are included in a multivariate logistic regression model to explore their associations with EC. Given that the average age of menopause for women in our country is 49.5 years, the age of 50 years is used as the cutoff in this study. Based on these analyses, a nomogram prediction model is constructed and internally validated. To further interpret the predictive mechanism of the established model, SHAP (SHapley Additive exPlanations) analysis was performed using the Python SHAP package (version 0.44.0), which quantifies the contribution and directionality of each predictor in the model.

## Results

The characteristics of the study population are shown in **Table 1**. There were no significant differences in education level, residence, hyperlipidemia, coronary heart disease, and number of pregnancies between the two groups (*P* > 0.05). The proportions of patients with age ≥ 50 years old, BMI ≥ 30 kg/m^2^, hypertension, diabetes, age at menopause > 50 years old, family history, and endometrial thickness ≥ 7 mm in the observation group were higher than those in the control group (*P* < 0.05).

**Table 1:** Descriptive statistics of the study population characteristics.

Indicators	Control group (n=475)	Observation group (n=89)	*χ* ^2^	*P* value
Age, years			16.421	< 0.001
< 50	239 (50.32)	24 (26.97)		
≥ 50	236 (49.68)	65 (73.03)		
BMI, kg/m^2^			92.031	< 0.001
< 30	380 (80.00)	27 (30.34)		
≥ 30	95 (20.00)	62 (69.66)		
Educational level			0.978	0.323
Junior high school and below	181 (38.11)	29 (32.58)		
Junior high school or above	294 (61.89)	60 (67.42)		
Place of residence			0.005	0.946
Village	210 (44.21)	39 (43.82)		
Towns and cities	265 (55.79)	50 (56.18)		
Hypertension			78.542	< 0.001
No	391 (82.32)	34 (38.20)		
Yes	84 (17.68)	55 (61.80)		
High blood lipids			0.009	0.926
No	420 (88.42)	79 (88.76)		
Yes	55 (11.58)	10 (11.24)		
Diabetes			45.061	< 0.001
No	444 (93.47)	62 (69.66)		
Yes	31 (6.53)	27 (30.34)		
Coronary heart disease			1.413	0.235
No	374 (78.74)	65 (73.03)		
Yes	101 (21.26)	24 (26.97)		
Menarche, year			0.822	0.365
< 12	242 (50.95)	50 (56.18)		
≥ 12	233 (49.05)	39 (43.82)		
Number of pregnancies			1.089	0.580
≤ 1	284 (59.79)	49 (55.06)		
2-3	138 (29.05)	27 (30.34)		
≥ 4	53 (11.16)	13 (14.61)		
Age at menopause			129.897	< 0.001
≤ 50	387 (81.47)	20 (22.47)		
> 50	88 (18.53)	69 (77.53)		
Family History			21.501	< 0.001
No	475 (100.00)	85 (95.51)		
Yes	0 (0.00)	4 (4.49)		
Endometrial thickness, mm			31.646	< 0.001
< 7 mm	203 (42.74)	10 (11.24)		
≥ 7 mm	272 (57.26)	79 (88.76)		

**Figure 2:**
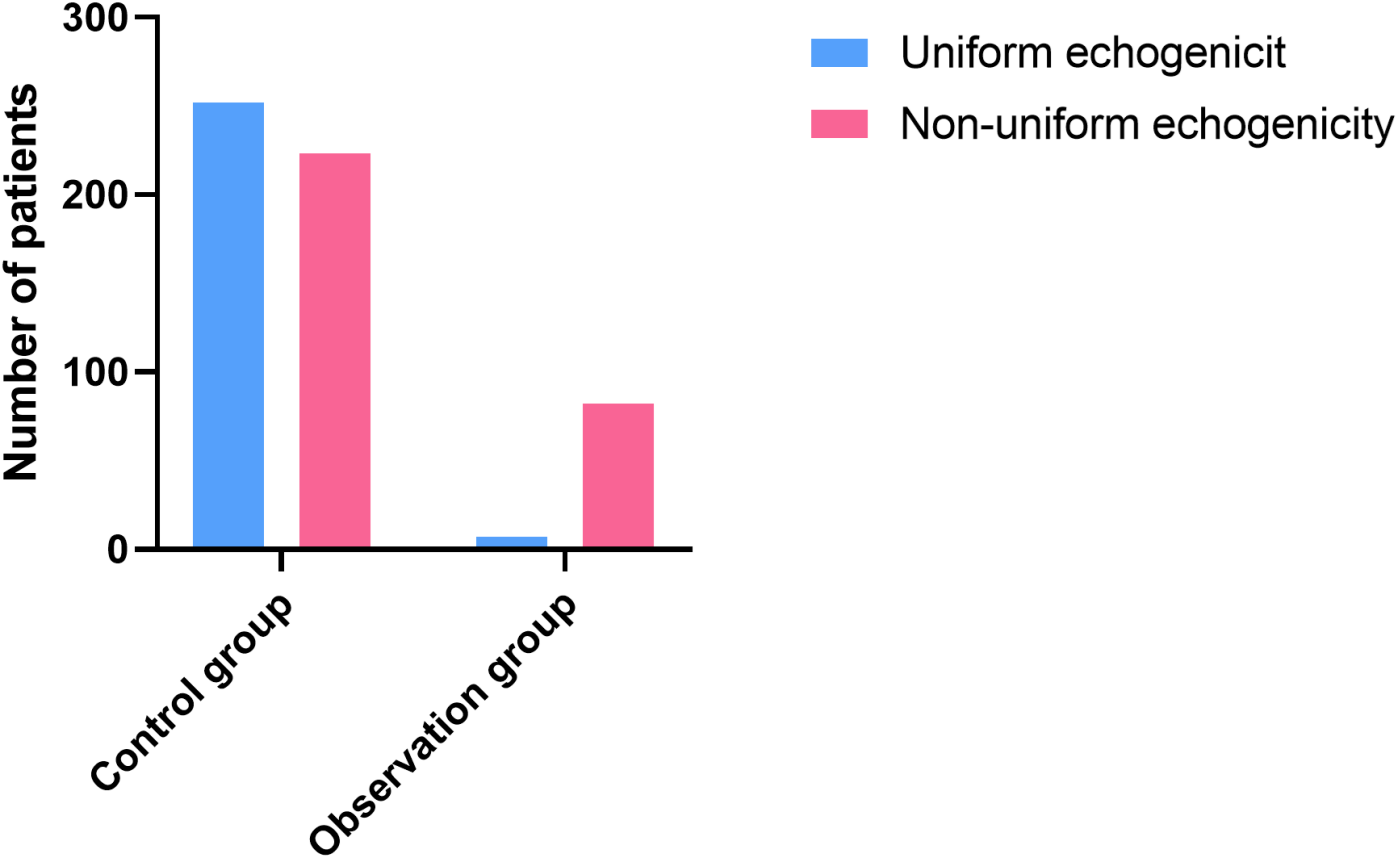
Endometrial echogenicity examined by TVS.

In the control group, 252 cases (53.05%) showed uniform echogenicity and 223 cases (46.95%) showed non-uniform echogenicity. In the observation group, 7 cases (7.87%) presented with uniform echogenicity, whereas 82 cases (92.13%) exhibited non-uniform echogenicity. The difference between the two groups was statistically significant (*P* < 0.05), as shown in **Figure 2**.

The sensitivity, specificity, accuracy, false positive rate and false negative rate of TVS detection of non-uniform echogenicity in the diagnosis of EC were 92.13%, 53.05%, 59.22%, 46.95% and 7.87%, respectively, as shown in **Table 2**.

**Table 2:** Comparison between the results of non-uniform echogenicity detection by TVS and pathological diagnosis.

Methods of diagnosis	Results of pathological diagnosis of EC	
	Positive (n=89)	Negative (n=475)
Non-uniform echogenicity		
Positive (n)	82	223
Negative (n)	7	252

**Figure 3:**
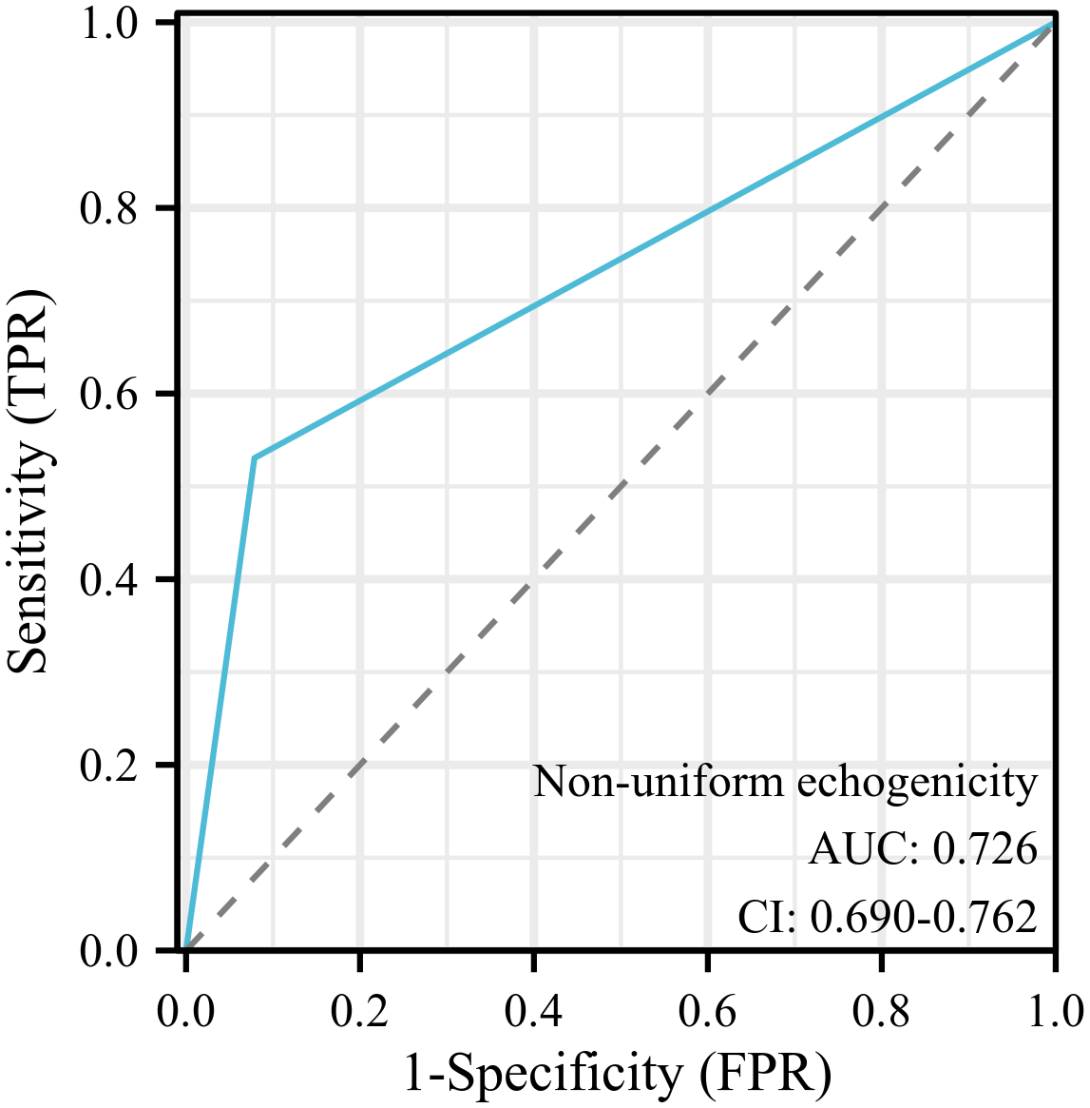
The ROC curve of EC endometrial echo was detected by transvaginal ultrasonography.

The ROC curve of vaginal ultrasound detection of non-uniform echogenicity in EC was drawn, and the results showed that vaginal ultrasound echo had certain diagnostic value in the predictive ability of EC (AUC = 0.726, 95% CI = 0.690-0.762), as shown in **Figure 3**.

Training set and validation set in age, BMI, hypertension, diabetes patients with indicators such as no statistical significance (*P* > 0.05), showing similar clinical features between training set and validation set, as shown in the **Table 3**.

**Table 3:** Training set and validation set single factor comparison.

Indicators		Training set (n=395)	Validation set (n=169)	*χ* ^2^	*P* value
Age, years	< 50	186 (47.09)	77 (45.56)	0.111	0.739
	≥ 50	209 (52.91)	92 (54.44)		
BMI, kg/m^2^	< 30	284 (71.90)	122 (72.19)	0.001	0.992
	≥ 30	110 (28.10)	47 (27.81)		
Hypertension	No	304 (76.96)	121 (71.60)	1.834	0.176
	Yes	91 (23.04)	48 (28.40)		
Diabetes	No	355 (89.87)	151 (89.35)	0.035	0.851
	Yes	40 (10.13)	18 (10.65)		
Age at menopause, years	≤ 50	285 (72.15)	122 (72.19)	0.000	0.993
	> 50	110 (27.85)	47 (27.81)		
Endometrial thickness, mm	< 7	147 (37.22)	66 (39.05)	0.170	0.680
	≥ 7	248 (62.78)	103 (60.95)		
Endometrial echogenicity	Uniform echogenicity	186 (47.09)	73 (43.20)	0.722	0.395
	Non-uniform echogenicity	209 (52.91)	76 (56.80)		

The statistically significant indicators in the study population characteristics were included in the multivariate Logistic regression analysis. BMI ≥30 kg/m^2^ (*OR*: 10.239, 95% *CI*: 0.828-5.092), hypertension (*OR*: 11.067, 95% *CI*: 4.185-29.263), diabetes (*OR*: 5.322, 95% *CI*: 1.392-20.343), age at menopause > 50 years (*OR*: 16.878 95% *CI*: 7.556-37.697), non-uniform echogenicity (*OR*: 9.879, 95% *CI*: 3.116-31.429) were risk factors for EC, as shown in **Table 4**.

**Table 4:** Multifactorial logistic regression analysis for EC.

Indicators		*β*	*OR*	95% *CI*	*P* value
Intercept		-7.872	0.000	0.000-0.003	< 0.001
Age, years	< 50				
	≥ 50	0.720	2.054	0.828-5.092	0.120
BMI	< 30				
	≥ 30	2.326	10.239	3.916-26.770	< 0.001
Hypertension	No				
	Yes	2.404	11.067	4.185-29.263	< 0.001
Diabetes	No				
	Yes	1.672	5.322	1.392-20.343	0.015
Age at menopause, years	≤ 50				
	> 50	3.008	20.243	7.752-52.861	< 0.001
Endometrial thickness, mm	< 7 mm				
	≥ 7 mm	0.714	2.042	0.706-5.905	0.118
Endometrial echogenicity	Uniform echogenicity				
	Non-uniform echogenicity	2.292	9.879	3.116-31.429	< 0.001

**Figure 4:**
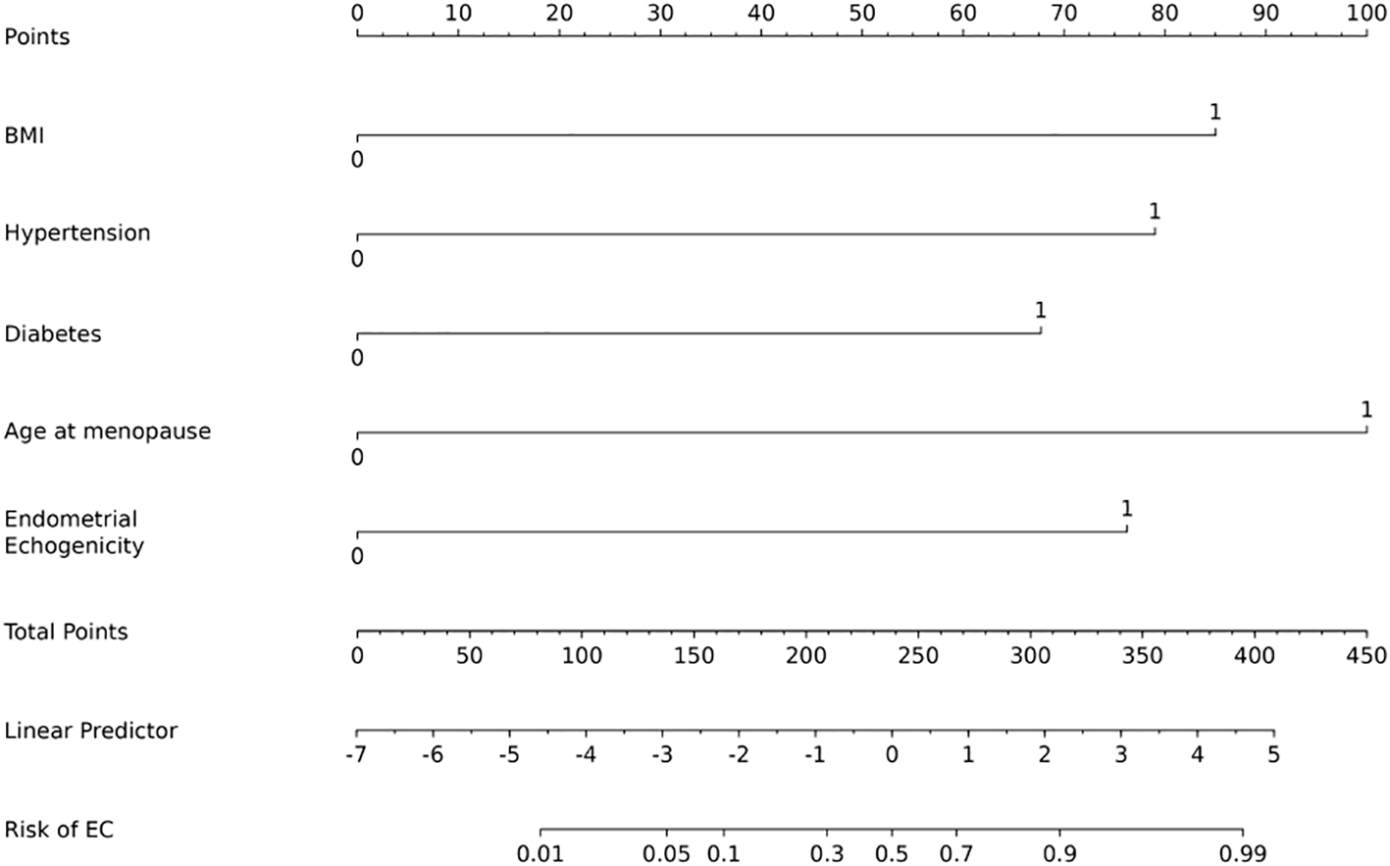
Nomogram prediction model for EC risk.

Based on the results of the multivariate analysis, a nomogram for predicting EC risk was constructed using age, BMI, hypertension, diabetes, age at menopause, endometrial thickness, and endometrial echogenicity as independent variables, with corresponding scores assigned to each predictor (**Figure 4**).

**Figure 5:**
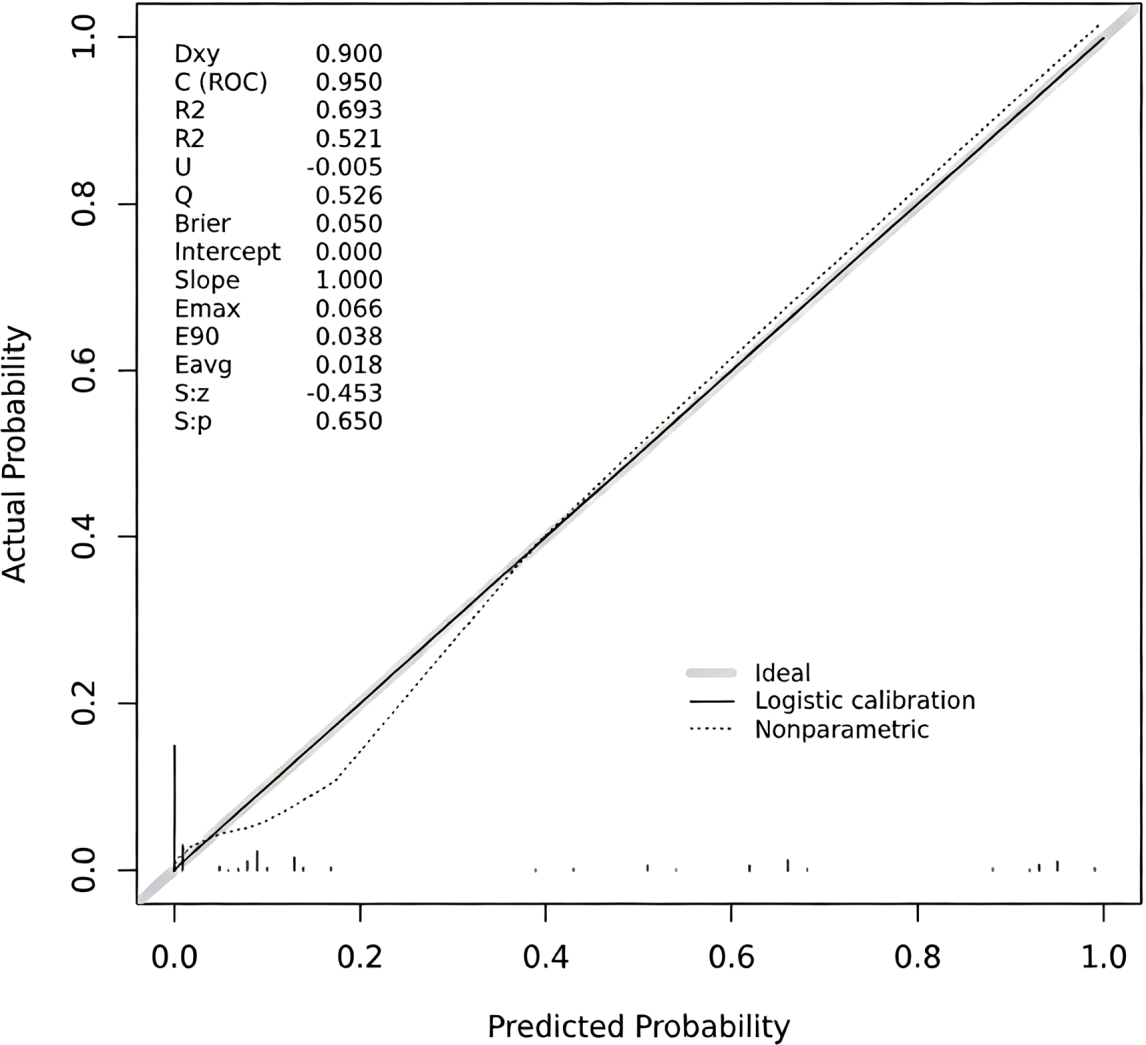
Model validation of the training set: Calibration curve.

**Figure 6:**
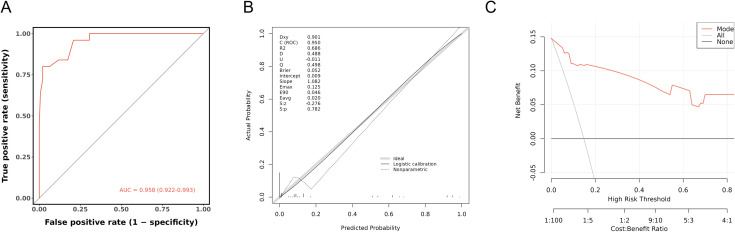
Model validation for the internal validation set. **(A)** ROC curve. **(B)** Calibration curve. **(C)** Decision curve analysis curve.

The nomogram model exhibited good calibration performance in the training set, with the calibration curve closely overlapping the ideal curve, indicating high consistency between predicted and actual risks (**Figure 5**). In the internal validation set, the model demonstrated excellent discrimination ability, with an AUC reaching 0.958, which is higher than the conventional threshold of 0.7, indicating its high predictive accuracy. Moreover, the calibration curve in the validation set also closely matched the ideal curve, further confirming the model’ s stable predictive consistency across different datasets. The results of decision curve analysis indicated that the model could provide significant clinical net benefit across a wide range of threshold probabilities, suggesting its good potential for clinical application (**Figure 6**).

**Figure 7:**
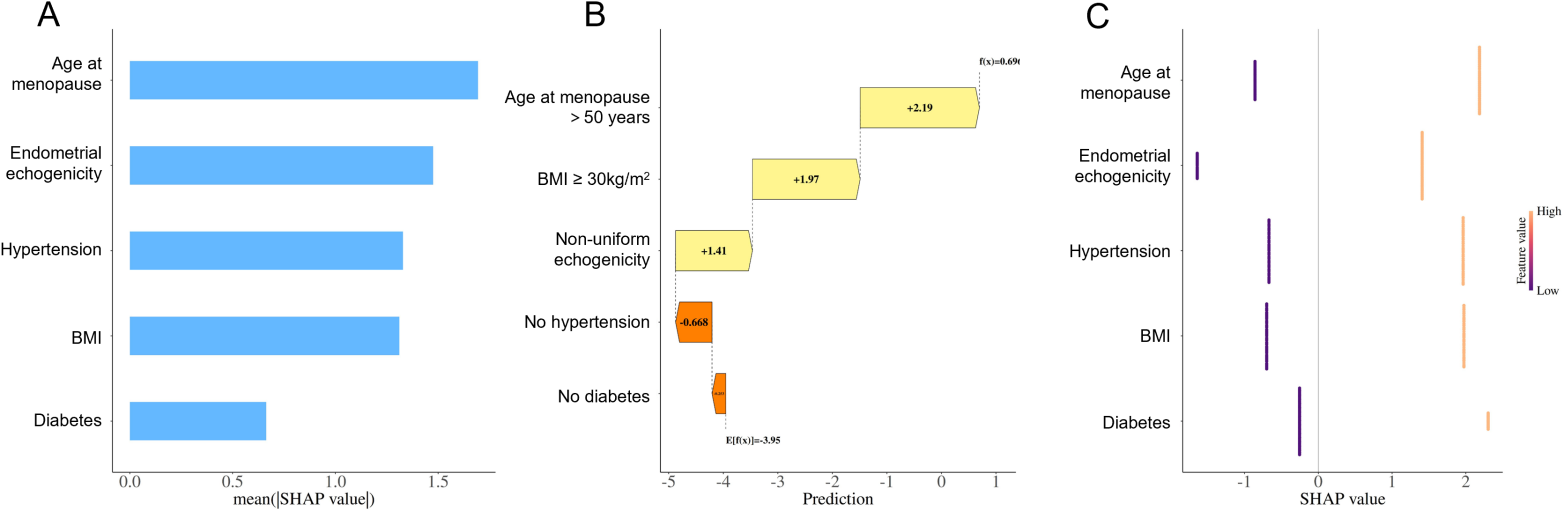
SHAP model validation. **(A)** Feature importance plot. **(B)** Single-sample waterfall plot. **(C)** Beeswarm plot of model features.

To further interpret the predictive mechanism of the constructed nomogram, SHAP interpretability analysis was performed. The feature importance plot (**Figure 7A**) revealed that age at menopause and endometrial echogenicity heterogeneity were the most influential predictors in the model. The beeswarm plot (**Figure 7B**) further showed that higher values of echogenicity heterogeneity were associated with SHAP values concentrated in the positive region, suggesting a stable positive association with EC risk that persisted after adjusting for other confounding factors. The single-sample waterfall plot (**Figure 7C**) visually illustrated the decomposition of predicted risk for individual patients, highlighting the model’s interpretability in personalized assessment.

**Figure 8:**
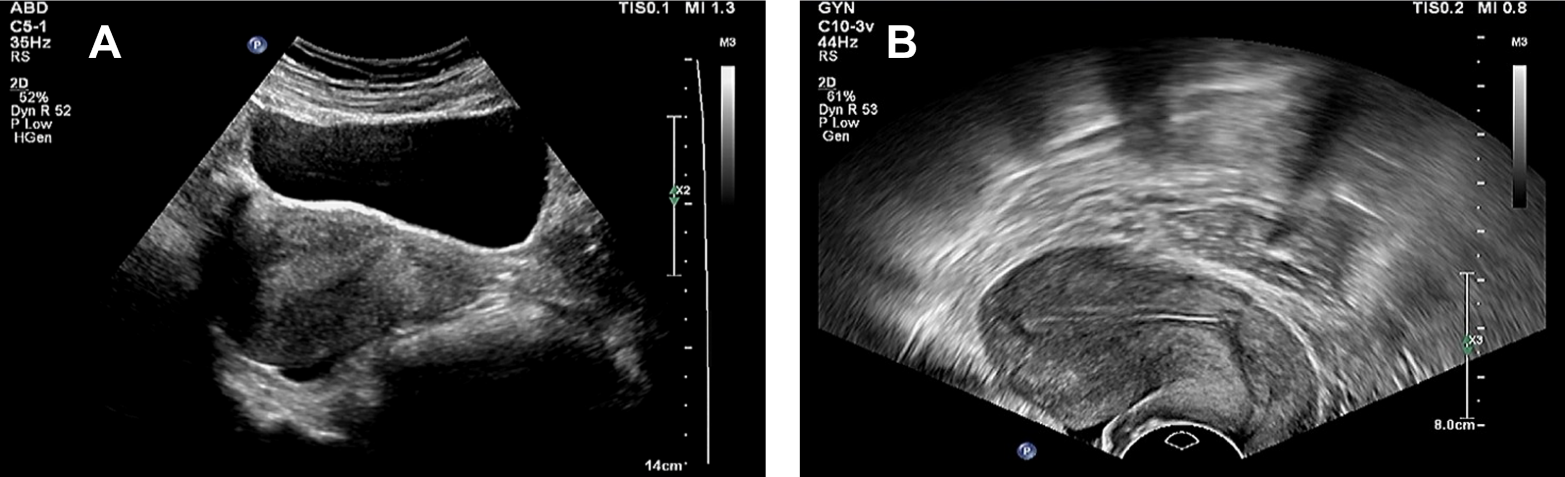
Endometrial ultrasound images of typical cases. **(A)** Heterogeneous echo (EC patient. **(B)** Homogeneous echo (patients with benign lesions).

To visually demonstrate the clinical utility of the nomogram, we first compared the predicted EC risk probability distribution from the model with actual outcomes using **Table 5**. The results showed that when the nomogram-predicted EC probability was ≥ 0.5, the number of cases was 93 (16.49%), whereas the actual number of confirmed EC cases was 89 (15.78%). There was no statistically significant difference between the two (*P* > 0.05), indicating that the model had good calibration. To further illustrate the application of the nomogram in individualized assessment, we selected the following two representative cases from the validation set for demonstration (**Figure 8**).

**Table 5:** EC risk probability score based on the nomogram.

Total points	Probability of EC risk	Number (%)
312	0.9	39 (6.91)
290	0.8	4 (0.71)
268	0.7	9 (1.60)
252	0.6	27 (4.79)
239	0.5	14 (2.48)
222	0.4	4 (0.71)
210	0.3	0 (0)
188	0.2	0 (0)
154	0.1	123 (21.81)
135	0.05	344 (60.99)

Case A (Diagnosed EC): A 62-year-old postmenopausal patient presented with vaginal bleeding. TVS revealed heterogeneous endometrial echotexture (**Figure 8A**). Her clinical characteristics included a BMI of 32 kg/m², hypertension, diabetes mellitus, and a menopausal age of 52 years. Based on the nomogram (**Figure 4**), the scores were as follows: BMI (85 points), hypertension (79 points), diabetes (68 points), menopausal age (100 points), and heterogeneous echotexture (76 points), resulting in a total score of 408 points, corresponding to a predicted EC probability of >90%. The patient was subsequently diagnosed with endometrioid adenocarcinoma (FIGO stage IA) via hysteroscopic biopsy.

Case B (Benign Lesion): A 48-year-old patient underwent evaluation for irregular menstruation. TVS showed homogeneous endometrial echotexture (**Figure 8B**). The patient had no history of hypertension but had diabetes, with a BMI of 25.7 kg/m². According to the nomogram, the total score was 68 points, corresponding to an EC risk of < 5%. Pathological examination of the diagnostic curettage specimen revealed benign endometrial hyperplasia without atypia.

Informed consent was obtained from both patients. These findings demonstrate that the nomogram effectively integrates ultrasound features and clinical variables, enabling individualized and quantitative assessment of EC risk and providing an intuitive reference for clinical decision-making.

## Discussion

EC, as one of the three major gynecological malignancies with a rising global incidence, has become the most common malignancy of the female reproductive system, particularly in developed countries (1). Its typical clinical manifestations include postmenopausal vaginal bleeding or abnormal uterine bleeding in premenopausal women (12). But early symptoms are often atypical, posing challenges for early diagnosis. This study focused on the diagnostic value of TVS. Results showed that 92.13% of patients in the observation group exhibited heterogeneous endometrial echogenicity, significantly higher than the 46.95% in the control group. This suggests heterogeneous echogenicity may be an early warning sign of endometrial pathology with potential for EC screening. TVS demonstrated a sensitivity of 92.13%, specificity of 53.05%, and accuracy of 59.22% in detecting this heterogeneity, indicating its ability to identify EC to some extent. However, TVS carries risks of missed diagnosis (false-negative rate: 7.87%) and misdiagnosis (false-positive rate: 46.95%), particularly for early-stage EC or cases without clear structural changes. Missed diagnoses often occur with small lesions or those located deep within the endometrium. Misdiagnosis risk stems from other endometrial pathologies (e.g., hyperplasia, fibroids, or polyps) potentially sharing similar ultrasound appearances with EC (13). ROC curve analysis (AUC = 0.726) further supports the value of heterogeneous echogenicity for EC’s early diagnosis. Although direct studies on its application in EC are relatively limited, its potential value is still highlighted by the promising applications in other fields, such as assessing the chemotherapy response in breast cancer (14) and diagnosing muscle disorders (15).

In terms of pathogenesis, the occurrence of EC is closely related to hormonal imbalance (16), with the continuous stimulation of estrogen in the absence of adequate progesterone antagonism being the main risk factor (17). Endometrium is precisely regulated by estrogen and progesterone in the menstrual cycle (18): estrogen dominates the growth of endometrium in the proliferative phase (19), and progesterone promotes the secretory phase transformation with estrogen. In the absence of pregnancy, hormone withdrawal initiates menstruation, during which estrogen completes endometrial regeneration by driving cell mitosis and differentiation (20). Long-term estrogen exposure or imbalance will lead to excessive endometrial proliferation, significantly increase the risk of gene mutation and abnormal differentiation, and eventually induce EC (21) Progesterone antagonizes its effect by down-regulating the concentration of estrogen receptor. When progesterone is relatively insufficient, the endometrium cannot complete the transformation into the secretory phase and retains the proliferative state, which directly increases the risk of EC (22). “Therefore, any factor that increases or enhances estrogen levels may increase EC susceptibility.” It is worth noting that, driven by lifestyle changes and prolonged menopause and other factors, the incidence of EC has continued to increase and become younger (23). This study thus focused on five EC risk factors: BMI, hypertension, diabetes, age at menopause > 50 years, and non-uniform echogenicity. Among them, BMI has been identified as a key risk factor -- epidemiological studies have confirmed that the risk of EC in overweight and obese women is 2.45 times and 3.5 times that of normal weight women respectively (24), and the mortality rate of obese patients is 6.2 times higher than that of non-obese patients (25). Obesity promotes EC mainly through three pathways: the conversion of androgen to estrogen through the aromatization of adipose tissue, which continuously stimulates pathological hyperplasia of the endometrium (26); It induces systemic low-grade inflammation through the release of adipokines, promotes the generation of ROS and reactive nitrogen species (RNS), and leads to DNA damage (27). And metabolic abnormalities exacerbate oxidative stress, excessive free radicals damage cellular structure and interfere with gene homeostasis (28).

Diabetes mellitus is an important risk factor for EC. Although it is not directly carcinogenic, it indirectly increases the risk of EC through its close association with obesity, metabolic syndrome, insulin resistance and chronic inflammation (29, 30). Insulin resistance, as the core mechanism, induces compensatory hyperinsulinemia and promotes EC through three pathways: 1) activation of PI3K/AKT and other pro-proliferative pathways leading to endometrial dysplasia; 2) inhibition of apoptosis and accumulation of abnormal cells; 3) stimulate the expression of vascular endothelial growth factor (VEGF) to induce tumor angiogenesis (31). The American Nurses’ Health Study (NHS) revealed the key rule: patients with diabetes duration <2 years had the highest risk of EC, while the risk of long-term patients was not significantly increased, suggesting that early metabolic disorders had a more significant impact on the intima (32). Hypertension also constitutes the risk of EC, which is mainly mediated by metabolic-hormone imbalance (33): 1) Insulin resistance, which is often accompanied by insulin resistance, increases free estrogen by activating IGF-1 receptor and reducing sex hormone binding globulin (SHBG), and promotes intimal hyperplasia (34); 2) Under the synergistic effect of obesity, the aromatic androgen in adipose tissue is changed to estrogen (35); 3) Excessive activation of renin-angiotensin system (RAS) induces oxidative stress and pathological angiogenesis through angiotensin II (Ang II) (36). It should be noted that some antihypertensive drugs (such as thiazide diuretics) may affect the risk through glucose metabolism, but further verification is needed.

Multivariable logistic regression in the present study showed that endometrial thickness is not an independent risk factor for EC, a finding that diverges from some earlier reports (37, 38). This discrepancy may be explained as follows. First, endometrial thickness is a highly variable morphological parameter with limited diagnostic specificity; benign conditions such as endometrial hyperplasia, polyps or submucosal myomas can all produce marked thickening, markedly diluting its weight in distinguishing malignant from benign disease. Second, after entering more specific ultrasonographic features (e.g., heterogeneous echogenicity, focal lesions, or an indistinct endometrial–myometrial junction) and clinical variables into the model, the independent predictive value of endometrial thickness was substantially attenuated. This suggests that the malignant biology of EC is reflected more directly by structural disruption manifesting as echotexture abnormalities than by simple thickness increments. Consequently, clinical practice should move away from reliance on a single endometrial thickness cut-off and instead adopt a multidimensional approach—integrating sonomorphological characteristics with menopausal status, bleeding history and other clinical data—to more accurately estimate EC risk.

In this study, a nomogram model incorporating five predictive factors was constructed based on the results of multivariate logistic regression analysis. In the training set, the model demonstrated excellent calibration, with the calibration curve closely overlapping with the ideal curve, indicating a high degree of consistency between predicted and actual risks. In the internal validation set, the model exhibited superior discriminatory ability, with an AUC as high as 0.960. It should be noted that this high AUC value may be partly attributable to the relatively low event rate in the current cohort, which could introduce a risk of overfitting and may explain the exceptionally high discrimination performance. SHAP interpretability analysis further revealed that age at menopause and heterogeneous endometrial echogenicity were the two most influential predictors in the model, with their SHAP value distributions indicating stable and consistent positive associations with EC risk. Despite this consideration, the calibration curve in the validation set remained closely aligned with the ideal curve, further confirming the model’s stable predictive consistency across different datasets. Decision curve analysis showed that the model provided significant clinical net benefit across a wide range of threshold probabilities, indicating its promising potential for clinical application. Notably, when the nomogram-predicted probability of EC was ≥ 0.5, the model identified 93 positive cases (16.49%), showing no statistically significant difference from the 89 actually confirmed cases (15.78%), further validating the consistency between the model’s predictions and actual clinical outcomes. Representative clinical cases also demonstrated high concordance between the nomogram predictions and subsequent pathological diagnoses. These findings indicate that the nomogram not only exhibits robust statistical predictive performance but also serves as an intuitive and interpretable clinical tool for individualized risk assessment and decision support in EC. In conclusion, the model developed in this study demonstrates clear clinical relevance, with SHAP analysis enhancing the interpretability of its decision-making mechanism, making it a promising tool for early identification and stratified management of individuals at high risk for EC.

Despite evaluating the role of ultrasonic echo heterogeneity in the early diagnosis of EC and exploring multiple risk factors in this study, several limitations should be acknowledged. First, the sample size of the case group (EC, n = 89) was considerably smaller than that of the control group (n = 475). Although this imbalance partly reflects the differential prevalence of the disease in the screening population, it may still affect the statistical power and generalizability of the predictive model, representing a major limitation of this study. Second, the retrospective design may introduce selection bias, especially in the analysis of family history data, as the availability and accuracy of such information could compromise the reliability of the results. Third, although a range of risk factors was included, future studies could incorporate additional or more granular variables, such as genetic markers and detailed hormonal profiles. Nevertheless, TVS remains an important screening tool for EC, especially for initial screening and follow-up, due to its non-invasive, cost-effective, and easily accessible nature, despite the aforementioned limitations. To further improve diagnostic accuracy and reduce false-positive and false-negative results, future research could focus on two main directions: First, integrating multimodal imaging techniques, such as 3D TVS, which can provide coronal imaging of the uterine cavity and more objective volumetric data and has been proven to be superior to traditional two-dimensional TVS in assessing endometrial morphology and the extent of lesions (39, 40). Second, combining TVS with serum biomarkers. These approaches could ultimately optimize the clinical diagnosis and treatment pathway for EC.

## Data Availability

The original contributions presented in the study are included in the article/supplementary material. Further inquiries can be directed to the corresponding author.

## Glossary

EC: Endometrial Cancer
TVS: Transvaginal Ultrasound
ROC: Receiver Operating Characteristic
AUC: Area Under The Curve
IETA: International Endometrial Tumor Analysis
BMI: Body Mass Index
DCA: Decision Curve Analysis
ANOVA: One-Way Analysis Of Variance
RNS: Reactive Nitrogen Species
VEGF: Vascular Endothelial Growth Factor
NHS: Nurses’ Health Study
SHBG: Sex Hormone Binding Globulin
RAS: Renin-Angiotensin System
Ang II: Angiotensin Ii
